# Perceived future career prospects in general practice: quantitative results from questionnaire surveys of UK doctors

**DOI:** 10.3399/bjgp16X687025

**Published:** 2016-08-31

**Authors:** Trevor W Lambert, Fay Smith, Michael J Goldacre

**Affiliations:** UK Medical Careers Research Group, Unit of Health-Care Epidemiology, Nuffield Department of Population Health, University of Oxford, Oxford.; UK Medical Careers Research Group, Unit of Health-Care Epidemiology, Nuffield Department of Population Health, University of Oxford, Oxford.; UK Medical Careers Research Group, Unit of Health-Care Epidemiology, Nuffield Department of Population Health, University of Oxford, Oxford.

**Keywords:** career choice, general practice, job satisfaction, primary care, secondary care

## Abstract

**Background:**

There are more studies of current job satisfaction among GPs than of their views about their future career prospects, although both are relevant to commitment to careers in general practice.

**Aim:**

To report on the views of GPs compared with clinicians in other specialties about their future career prospects.

**Design and setting:**

Questionnaire surveys were sent to UK medical doctors who graduated in selected years between 1974 and 2008.

**Method:**

Questionnaires were sent to the doctors at different times after graduation, ranging from 3 to 24 years.

**Results:**

Based on the latest survey of each graduation year of the 20 940 responders, 66.2% of GPs and 74.2% of hospital doctors were positive about their prospects and 9.7% and 8.3%, respectively, were negative. However, with increasing time since graduation and increasing levels of seniority, GPs became less positive about their prospects; by contrast, over time, surgeons became more positive. Three to 5 years after graduation, 86.3% of those training in general practice were positive about their prospects compared with 52.9% of surgical trainees: in surveys conducted 12–24 years after graduation, 60.2% of GPs and 76.6% of surgeons were positive about their prospects.

**Conclusion:**

GPs held broadly positive views of their career prospects, as did other doctors. However, there was an increase in negativity with increasing time since graduation that was not seen in hospital doctors. Research into the causes of this negativity and policy measures to ameliorate it would contribute to the continued commitment of GPs and may help to reduce attrition.

## INTRODUCTION

GP recruitment in the UK is in crisis.^1^ Greater patient demand, an ageing population profile, an increasing population coupled with the switching of the management of chronic conditions from secondary to primary care, and an ageing GP workforce, have led to an increased need for more doctors to train in general practice.^2^ However, many existing training posts have gone unfilled^1^ and the expansion of training schemes alone is unlikely to have the desired outcome. Other areas of concern include increasing numbers of females in their 30s leaving general practice,^1^ and high workload.^3^^,^^4^ In a survey of GPs conducted in 2015, 71% said that high workload negatively affected their personal commitment to general practice.^3^

Previous research among GPs has found that low job satisfaction is associated with higher levels of intentions to leave medicine.^5^ A study of American physicians found that those who were very dissatisfied with their jobs were twice as likely to retire early as those who were satisfied, and were over three times more likely to reduce their hours to <20 hours per week before retirement.^6^ Job satisfaction is higher in individuals who perceive their job to have better career prospects.^7^

The aim of this study was to report the views of GPs regarding their perceived career prospects and to compare GPs’ views with those of other medical practitioners, with doctors who qualified at different times, and with doctors who were at different stages of their careers at the time of survey.

## METHOD

The UK Medical Careers Research Group (MCRG) has surveyed all UK medical graduates from nine year-of-graduation cohorts: 1974, 1983, 1988, 1993, 1996, 1999, 2000, 2002, and 2008. Doctors were surveyed at different times after graduation ranging 3–24 years, in surveys which took place between 1996 and 2013. Some cohorts were surveyed more than once. The MCRG used postal and email questionnaires and up to four reminders were sent to non-responders. Further details of the methodology are available elsewhere.^8^

In each of 19 surveys conducted over a 17-year period, among other questions the MCRG asked doctors to rate their level of positivity about their career prospects. In 12 of the surveys this was done by rating agreement with the following statement: ‘My career prospects are good’ (the statement offered a 5-point scale covering ‘strongly agree’, ‘agree’, ‘neither agree nor disagree’, ‘disagree’, and ‘strongly disagree’, with a ‘no opinion’ option). Occasionally the authors used ‘My future career prospects are good’, and in seven surveys they used the wording ‘I am satisfied with my future career prospects’, with the same response rating options. The responses were analysed on the 5-point scale, and re-analysed on a 3-point scale (with ‘strongly agree’ and ‘agree’ combined, and ‘strongly disagree’ combined with ‘disagree’).

**How this fits in**

Previous research among GPs has found that low job satisfaction is associated with higher levels of intentions to leave medicine. In this context the authors decided to compare the views of GPs and other doctors about their career prospects, using data from the UK Medical Careers Research Group studies collected over many years. Most GPs were positive about their prospects. However, the level of negativity among GPs about their future career prospects increased with increasing seniority: this was not seen in hospital doctors.

To avoid possible confusion with job satisfaction, the authors refer in the analysis to agreement or disagreement that ‘My career prospects are good’.

In all, the authors surveyed 37 332 UK doctors from nine different cohorts. The statement about future prospects was included in surveys of the cohorts of 1974, 1983 and 2008 on one occasion, those of 1988, 1999, 2000, and 2002 twice, the cohort of 1996 three times, and the cohort of 1993 five times. The cohorts ranged in size from 2347 graduates in 1974 to 6795 graduates in 2008.

The authors investigated the doctors’ level of agreement that their career prospects were good. In particular the authors were interested in whether doctors working in general practice differed from doctors working in other specialties. Doctors’ descriptions of their specialty of working were grouped by the authors into 13 specialty groups comprising: adult hospital medical specialties, paediatrics, emergency medicine, hospital surgical specialties, obstetrics and gynaecology, anaesthetics, radiology, clinical oncology, pathology, psychiatry, general practice, community health, and public health.

The authors were also interested in variation in agreement or disagreement about career prospects by time since graduation, by sex, and by year when surveyed.

Responses were compared using χ^2^ tests for two-sample comparisons (reporting Yates’s continuity correction where there was only one degree of freedom). The authors used binary logistic regression to analyse the effect of factors in combination. Regression analyses used only those variables which were significant as single variables. For convenience of analysis, time since graduation was grouped into 3–5 years, 6–11 years, and 12–24 years; survey year was grouped into 1996 to 1999, 2000 to 2005, and 2010 to 2013; and specialty group for some analyses was further aggregated into four specialty groups: (adult) hospital medical specialties, hospital surgical specialties, general practice, and other hospital specialties combined (paediatrics, emergency medicine, obstetrics and gynaecology, anaesthetics, radiology, clinical oncology, pathology, and psychiatry).

## RESULTS

### Response rates

The aggregated response rate from contactable doctors, over all 19 surveys, was 68.3% (47 274/69 264; Appendix 1). Of the 47 274 survey responses, 8.4% (3956) answered ‘no opinion’ or did not select any of the possible responses to the question about career prospects. For the rest of this study, these were omitted, giving a total of 43 318 responses on the 5-point scale.

### Results on career prospects, using the latest data from each graduation year

Combining all responses from the latest survey of each graduation year, comprising nine surveys (20 940 responders), 14.1% of GPs strongly agreed that their future prospects were good, 52.1% agreed, 24.1% neither agreed nor disagreed, 7.7% disagreed, and 2.0% strongly disagreed (Table 1). For convenience, for the rest of the results, percentages are reported on a 3-point scale.

**Table 1.** Percentage agreement with future career prospects statement, by specialty grouping and sex, for the latest survey of each graduation year^a,b^

**Sex and specialty grouping**	**Statement: My future career prospects are good^c^**					

	**Strongly agree, %**	**Agree, %**	**Neither agree nor disagree, %**	**Disagree, %**	**Strongly disagree, %**	**Total, *n* (%)**
**Males and females**						
GPs	14.1	52.1	24.1	7.7	2.0	6939 (100)
Hospital doctors	18.0	56.2	17.6	6.5	1.8	10 943 (100)

**Males**						
GPs	15.6	48.7	24.6	8.2	2.9	2995 (100)
Hospital doctors	20.5	56.5	16.4	5.2	1.5	6097 (100)

**Females**						
GPs	13.0	54.7	23.7	7.4	1.3	3944 (100)
Hospital doctors	14.8	55.8	19.0	8.2	2.1	4846 (100)

a Includes results from the following surveys; 1974 and 1983 cohorts in 1998; 1996 cohort in 2003; 1988 cohort in 2004; 1993 cohort in 2010; 1999 and 2000 cohorts in 2012; 2002 and 2008 cohorts in 2013.

b Excludes doctors who were working in non-medical jobs or were not in paid employment.

c See Method for details of wording variation in the statement in some surveys.

The GPs were less positive about their prospects than were the hospital doctors (*z* = −11.2, *P*<0.001) and showed 66.2% agreement that prospects were good compared with 74.2% for the hospital doctors. A larger percentage of responses from GPs showed uncertainty about prospects (24.1%) than was the case for the hospital doctors (17.6%). A negative view of prospects was a little higher among GPs (9.7%) than among hospital doctors (8.3%).

Female GPs were marginally less positive about their prospects than female hospital doctors (*z* = −2.6, *P* = 0.01; 67.7% satisfied compared with 70.6%). Among males the difference was larger: 64.3% of GPs and 77.0% of hospital doctors were positive (*z* = −11.9, *P*<0.001). Appendix 2 shows the aggregated results for all surveys. The overall results for each of the 19 surveys are shown in Appendix 3.

### Statistical analysis of 15 surveys

#### Balance

In further analysis, the authors wanted to use more of the available 19 surveys, while ensuring that the most frequently surveyed cohorts, those of 1993 and 1996, did not contribute disproportionately to the analysis. The authors omitted four surveys of the 19 undertaken; see Appendix 4 for details. This enabled a balance to be achieved when considering results by year of survey and by years since graduation, whereby the 1996 to 1999 year of survey group featured the graduates of 1974, 1983, 1988, 1993, and 1996 once each, the 2000 to 2005 group featured the graduates of 1988, 1996, 1999, 2000, 2002, and 1993 once each, and the 2010 to 2013 group featured the graduates of 1993, 1999, 2000, 2002, 2008 once each. When considered by years since graduation, the 15 surveys comprised at 3–5 years after graduation the graduates of 1996, 2000, 2002, 2008 once each; at 6–11 years the graduates of 1988, 1993, 1996, 1999, and 2002 once each; and at 12–24 years the graduates of 1974, 1983, 1988, 1993, 1999, and 2000 once each.

### Subgroups

Within the 15 surveys considered here, there were statistically significant differences in the percentages of those who were positive about their future prospects (that is, those who strongly agreed or agreed that their prospects were good) when comparing groups by each of specialty group, survey year, number of years since graduation, and sex: see the univariable analysis results in Table 2. However, the percentage differences between the subgroups were modest. When all factors were entered into a logistic regression model simultaneously (Table 2; Multivariable analysis) each factor remained significant. GPs showed a slightly higher level of agreement that their prospects were good, compared with surgeons and those in the hospital medical specialties (Table 2). There was no evident trend across the three periods of survey year or years since graduation. Re-analysis with ‘disagreement’ as the criterion (Appendix 5) gave very similar results.

**Table 2.** Agreement that future prospects were good: UK medical graduates by years after graduation, sex, survey year, and specialty

**Predictor**	**Group**	**Agreement that prospects were good**		**Univariate analysis^a^**			**Multivariate analysis**	
		
		**%**	**n/N**	**df**	**χ^2^**	***P*-value**	**Wald**	***P*-value**
Specialty	General practice	70.5	7233/10 259					
	Hospital medical specialties	69.8	3818/5467	3	88.9	<0.001	104.6	<0.001
	Surgery	68.1	2755/4048					
	Other hospital	74.6	8252/11 057					

Sex	Males	72.3	10 853/15 009	1	7.6	<0.01	48.3	<0.001
	Females	70.8	11 205/15 822					

Years after graduation	3–5	70.2	6022/8579	2	234.3	<0.001	268.4	<0.001
	6–11	76.8	8068/10 502					
	12–24	67.8	7968/11 750					

Survey year	1996 to 1999	65.1	6881/10 566	2	381.2	<0.001	437.6	<0.001
	2000 to 2005	77.1	8323/10 799					
	2010 to 2013	72.4	6854/9266					

‘Univariable’ denotes single factor χ^2^ test for each predictor. ‘Multivariable’ denotes binomial logistic regression result for each predictor with all other predictors in the model. Cases are excluded where one or more predictors were missing, which reduced the sample size from 37 229 to 30 541.

a The univariable analysis here refers to the prospects variable with three levels: agree, neither, disagree. df = degrees of freedom.

#### Sex differences

There were modest differences in the views about career prospects between the sexes. However, female GPs were more positive about their career prospects than male GPs, with 72.2% of females and 68.1% of males holding a positive view (χ^2^_1_ = 19.9, *P*<0.001). In the other specialty groups males were more positive than females (Table 3: χ^2^_1_ = 12.6, 14.6, 39.3 for surgery, medical specialties, and other hospital specialties respectively; all *P*<0.001).

**Table 3.** Percentage agreement with future career prospects statement, by sex and specialty

**Sex and specialty**	**Agreement, %**	**Neither, %**	**Disagreement, %**	**Total, *n* (%)**
**Females**				
GP	72.2	20.4	7.4	6078 (100)
Surgery	63.6	17.1	19.3	1041 (100)
Medical specialties	67.4	18.7	13.9	2721 (100)
Other hospital specialties	72.2	16.5	11.3	5982 (100)

**Males**				
GP	68.1	21.9	10.0	4181 (100)
Surgery	69.6	16.6	13.8	3007 (100)
Medical specialties	72.2	17.5	10.3	2746 (100)
Other hospital specialties	77.5	14.8	7.7	5075 (100)

#### Time since graduation

Much larger differences in views about prospects were found when comparing ‘time from graduation’ in different specialties.

**Figure 1.**
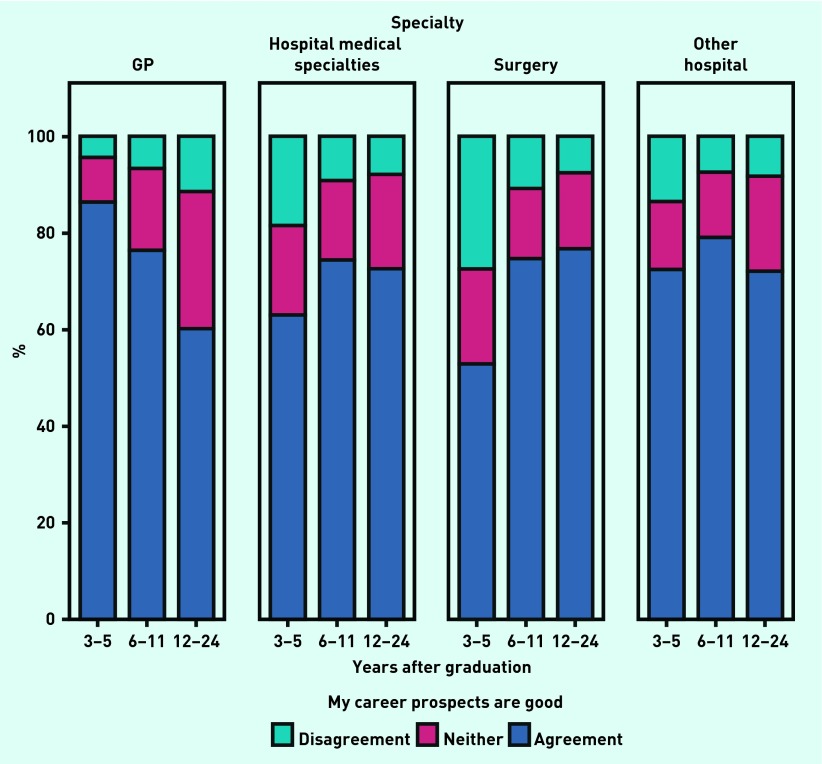
***Percentages of doctors who agreed that their future career prospects were good, at various stages (numbers of years) after graduation and by specialty group.***

GPs became less positive about their career prospects the longer they had worked since graduation, while for surgeons the reverse was true (Figure 1). Among doctors surveyed 3–5 years after graduation, GPs held more positive views than doctors in other specialty groups about their future career prospects (χ^2^_6_ = 514.2, *P*<0.001; Figure 1): for example, 86.3% of GPs were positive about their future career prospects compared with 52.9% of surgeons.

Comparing doctors surveyed 12–24 years after graduation, however, GPs were less likely than doctors working in other specialties to be positive about their future career prospects (χ^2^_6_ = 227.6, *P*<0.001): for example, 60.2% of GPs and 76.6% of surgeons were positive about their prospects.

#### Year of survey

**Figure 2.**
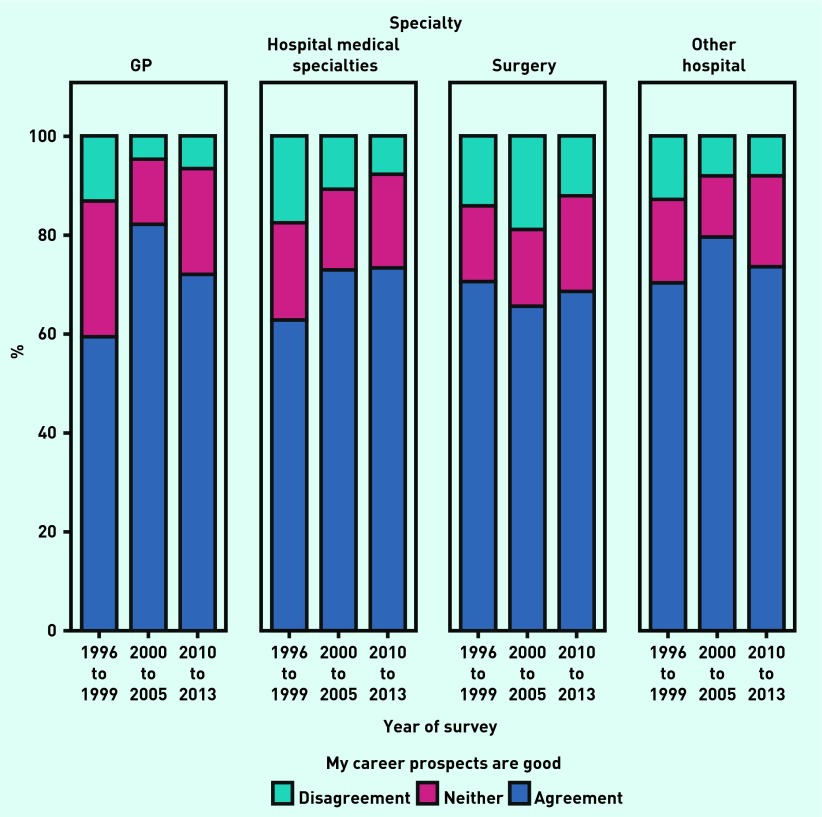
***Percentages of doctors who agreed that their future career prospects were good, at various survey years and by specialty group.***

For each of the four specialty groups, the level of agreement that career prospects were good, was compared across the three ‘year of survey’ periods (Figure 2). For GPs, those in the hospital medical specialties, those in surgery, and those in other hospital specialties, the year groups differed in their levels of agreement (χ^2^_4_ = 464.8, 132.7, 96.3 and 32.5 respectively; all *P*<0.001). GPs who were surveyed between 2000 and 2005 (82.1%) rated their prospects higher than GPs who were surveyed between 1996 and 1999 (59.3%) and those surveyed between 2010 and 2013 (72.1%). There was a similar peak between 2000 and 2005 for doctors in other hospital specialties (Figure 2).

### Comparison of perceived career prospects, comparing prospects at years 1 and 5 years after graduation

Doctors graduating in years 1999, 2000, and 2002 stated their views of their prospects in years 1 and year 5 and their responses were matched (Appendix 6).

Classifying responders by specialty based on their posts held in year 5, only 6.2% of GPs became more positive about their prospects by year 5, compared with 31.6% of surgeons, and 23.4% of GPs became more negative about their prospects by year 5, compared with 14.0% of surgeons (both Wilcoxon and marginal homogeneity tests gave *P*<0.001 in each case). This corresponds to the earlier findings of increasing positivity among surgeons and decreasing positivity among GPs, with increasing time since graduation (Figure 1).

## DISCUSSION

### Summary

Overall, most GPs were positive about their future career prospects, as were most doctors in hospital-based specialties. Compared with GPs, surgeons showed slightly lower levels of positivity and doctors working in hospital specialties other than medical specialties or surgery showed slightly higher levels. One-fifth of GPs were undecided about their prospects. Dissatisfaction with prospects was marginally higher among male GPs compared with male hospital doctors: for females the reverse was true.

GPs’ satisfaction with their prospects was lower among doctors surveyed later in their careers: for surgeons, the reverse was true. One possibility with some of the hospital specialties (perhaps notably with the surgical specialties) is that, in the early years, the doctors are concerned that they may not progress to senior posts within the specialty. Compared with the GPs at a similar stage, the specialists are still competing for career progression in terms of increasing seniority. As time progresses, and the individuals in the hospital specialties are successful, this in itself may increase their confidence about their future prospects.

Females working in hospital specialties were less likely than males in hospital specialties to be positive about their future career prospects. By contrast, female GPs were more positive about their future career prospects than male GPs.

Female GPs, like females working in the ‘other’ hospital specialties, were more positive about their future prospects than females working in hospital medical specialties and surgery. Males working in other hospital specialties were more likely than doctors working in the other specialties to agree that they were positive about their future career prospects.

### Strengths and limitations

The study was national and included all UK medical schools. It covered many graduation years from 1974 to 2008 and data were collected between 1996 and 2013. The response rates for each survey were good. The authors are an independent research group and they believe they receive honest answers from doctors. Due to the nature of survey research methodology there is the possibility of responder bias.

Some graduation cohorts were surveyed more often than others. To prevent any disproportionate contribution to the analysis from these cohorts, the authors used a balanced design, which means that in some of the analyses not all of the survey data were used.

### Comparison with existing literature

Most GPs were positive about their future career prospects. This tallies with other indicators of career motivation observed recently: 47% of GPs would recommend general practice as a career choice;^3^ 87% of GPs were satisfied with work and training, and 72% were satisfied with work–life balance.^9^

Female GPs were more positive about their future career prospects than male GPs; also, female GPs were more positive about their future prospects than females working in hospital medical specialties and surgery. Compatibility of general practice with family life is a factor.^10^ Female and male GPs may compare their situations to their peers in other specialties favourably, (in the case of some female GPs or unfavourably, (in the case of some male GPs).

Nearly one-quarter of GPs became more negative about their prospects by their fifth year after graduation. This could be due to a gradual realisation of the many challenges faced by GPs currently in terms of an ageing workforce, increasing workload, and increased patient demand.^2^^,^^3^ The latest data reported in this study were collected in 2013: in 2016, increasing pressures on service delivery suggest that this negative trend may continue.

### Implications for research and practice

Although the profile of views about career prospects was broadly positive for GPs, as it was for other doctors, there were some concerns. Specifically, the authors noted among GPs an increase in negative views of career prospects with increasing length of time since graduation, which is not seen in hospital doctors, and these changes were more noticeable among male GPs than among female GPs. Research into the causes of this negativity, and the introduction of policy measures to ameliorate its causes, would contribute to the continued commitment of GPs and may help to reduce attrition of senior GPs from the profession.

## Appendix 1. Number and percentage of responding doctors in the study, 1996 to 2013

**Table:**

**Year of survey**	**Cohort**	**Years after graduation**	**Cohort, *n***	**Contactable,^a^*n***	**Response *n*(%)**	**Answered question, *n***	**Did not answer/‘no opinion‘, *n***
1996	1993	3	3667	3517	2777 (79.0)	2482	295
1998	1974	24	2347	2138	1716 (80.3)	1633	83
	1983	15	3845	3651	2879 (78.9)	2621	258
1999	1988	11	3737	3570	2726 (76.4)	2503	223
	1993	6	3667	3560	2731 (76.7)	2590	141
	1996	3	3868	3777	2721 (72.0)	2593	128
2001	1996	5	3868	3674	2521 (68.6)	2287	234
2002	1993	9	3667	3451	2437 (70.6)	2406	31
2003	1996	7	3868	3682	2410 (65.5)	2377	33
2004	1988	16	3737	3493	2521 (72.2)	2452	69
	1993	11	3667	3490	2310 (66.2)	2197	113
2005	1999	6	4213	3988	2660 (66.7)	2417	243
	2000	5	4428	4120	2703 (65.6)	2485	218
	2002	3	4432	4239	2748 (64.8)	2297	451
2010	1993	17	3667	3470	2505 (72.2)	2389	116
2012	1999	13	4213	4030	2255 (56.0)	1207	1048^b^
	2000	12	4428	4200	2229 (53.1)	2147	82
2013	2002	11	4432	3196	2056 (64.3)	1960	96
	2008	5	6795	4018	2369 (59.0)	2275	94

Total			76 546	69 264	47 274 (68.3)	43 318	3956

a Excludes those with no address, deceased, not registered, or refusing to take part.

b Owing to a printing error, one mailing excluded the statement about career prospects.

## Appendix 2. Percentage agreement with future career prospects statement, by specialty and sex, for all 19 surveys combined

**Table:**

**Specialty**	**My career prospects are good**						

	**Strongly agree, %**	**Agree, %**	**Neither agree nor disagree, %**	**Disagree, %**	**Strongly Disagree, %**	**Total**	

						***n***	**%**
**All**							
GP	16.6	54.5	20.8	6.7	1.5	12 509	100
Hospital medical specialties	15.8	54.5	18.5	8.9	2.3	7150	100
Surgery	17.7	50.6	17.2	10.6	3.8	5234	100
Other hospital	17.5	57.7	15.6	7.3	1.9	14 315	100
Non-hospital medical	20.1	45.8	19.0	10.8	4.4	1280	100
Total	17.0	54.8	18.0	8.0	2.1	40 488	100

**Males**							
GP	17.4	51.7	21.5	7.4	2.1	5039	100
Hospital medical specialties	17.3	55.4	17.9	7.6	1.7	3633	100
Surgery	18.6	50.8	17.2	9.8	3.7	3905	100
Other hospital	20.2	57.8	14.7	5.6	1.6	6570	100
Non-hospital medical	27.3	44.5	16.8	8.6	2.7	512	100
Total	18.8	54.1	17.6	7.4	2.2	19 659	100

**Females**							
GP	16.0	56.4	20.3	6.2	1.1	7470	100
Hospital medical specialties	14.3	53.5	19.1	10.2	2.9	3517	100
Surgery	15.1	50.1	17.2	13.2	4.4	1329	100
Other hospital	15.3	57.6	16.3	8.8	2.1	7745	100
Non-hospital medical	15.2	46.6	20.4	12.2	5.5	768	100
Total	15.4	55.6	18.4	8.5	2.1	20 829	100

Note that this table excludes 2830 doctors who were working in non-medical jobs or were not in paid employment.

## Appendix 3. Percentage agreement with future career prospects statement: for all surveys

**Table:**

**Cohort**	**My career prospects are good**						

	**Survey year**	**Strongly Agree, %**	**Agree, %**	**Neither, %**	**Disagree, %**	**Strongly disagree, %**	**Total, *n***
1974	1998	20.0	35.7	27.4	11.8	5.1	1633
1983	1998	20.0	41.9	24.2	10.2	3.7	2621
1988	1999	23.5	45.8	19.8	8.3	2.7	2503
	2004	17.2	54.4	20.6	6.1	1.8	2452
1993	1996	8.9	60.3	23.3	6.5	1.0	2482
	1999	13.6	59.3	17.5	7.0	2.7	2590
	2002	15.9	55.3	18.2	8.6	2.0	2406
	2004	18.8	60.7	15.4	4.0	1.1	2197
	2010^a^	0.0	68.0	26.1	5.9	0.0	2389
1996	1999	9.4	49.7	17.5	18.4	4.9	2593
	2001	18.1	55.9	14.0	9.5	2.5	2287
	2003	19.9	63.0	11.6	4.8	.7	2377
1999	2005	26.1	55.7	10.6	6.2	1.4	2417
	2012	19.7	52.1	18.8	7.6	1.7	1207
2000	2005	26.3	51.1	13.7	6.8	2.1	2485
	2012	20.9	53.0	18.6	6.1	1.5	2147
2002	2005	10.0	57.3	13.1	14.1	5.4	2297
	2013	19.5	52.9	19.1	6.5	2.0	1960
2008	2013	14.3	59.5	15.6	8.0	2.6	2275

Total		16.8	54.6	18.0	8.3	2.4	43 318

This table excludes doctors who did not reply to the question or who selected ‘no opinion’.

a The 1993 in 2010 survey only offered three responses: Agree, Neither agree nor disagree, and Disagree.

## Appendix 4. Surveys included within this study

**Table:**

**Survey year**	**Years after graduation**								

	**3–5**			**6–11**			**12–24**		
		
	**1996–1999**	**2000–2005**	**2010–2013**	**1996–1999**	**2000–2005**	**2010–2013**	**1996–1999**	**2000–2005**	**2010–2013**
Surveys included	1993 in 1996	2002 in 2005	2008 in 2013	1993 in 1999	1999 in 2005	2002 in 2012	1983 in 1998	1988 in 2004	2000 in 2012
	1996 in 1999	1996 in 2001		1988 in 1999	1996 in 2003		1974 in 1998		1999 in 2012
		2000 in 2005		1993 in 2002					1993 in 2010
				1993 in 2004					

The surveys shown in shaded cells were not included in statistical analysis (see ’Balance’ section of Results).

## Appendix 5. Disagreement that future prospects were good: UK medical graduates by years after graduation, sex, survey year and specialty

**Table:**

**Predictor**	**Group**	**Disagreement that prospects were good**		**Univariate analysis^a^**			**Multivariate analysis**	
		
		**%**	**n/N**	**df**	χ**^2^**	***P*-value**	**Wald**	***P*-value**
Specialty	General practice	8.5	869/10 259					
	Hospital medical specialties	12.1	659/5467	3	161.8	<0.001	150.3	<0.001
	Surgery	15.2	617/4048					
	Other hospital	9.7	1069/11 057					

Sex	Male	10.1	1510/15 009	1	3.8	<0.05	26.2	<0.001
	Female	10.8	1704/15 822					

Years after graduation	3–5	14.9	1279/8579	2	266.9	<0.001	266.4	<0.001
	6–11	7.9	828/10 502					
	12–24	9.4	1107/11 750					

Survey year	1996–1999	13.9	1470/10 566	2	219.3	<0.001	304.7	<0.001
	2000–2005	9.2	993/10 799					
	2010–2013	7.9	751/9466					

‘Univariable’ denotes single factor χ^2^ test for each predictor. ‘Multivariable’ denotes binomial logistic regression result for each predictor with all other predictors in the model. Cases were excluded where one or more predictors were missing, which reduced the sample size from 37 229 to 30 541.

a The univariable analysis here refers to the prospects variable with two levels: agree versus neither and disagree combined. df = degrees of freedom.

## Appendix 6: Numbers of doctors who agreed and disagreed that their career prospects were good at 1 year and 5 years after graduation (1999, 2000, and 2002 graduation cohorts)

**Table:**

**Prospects at 1 year**	**Prospects at 5 years**			

	**Agree**	**Neither**	**Disagree**	**Total**
**GPs**				
Agree	**546**	153	32	731
Neither	37	**13**	4	54
Disagree	7	6	**8**	21
Total	590	172	44	806

**Hospital medical specialists**				
Agree	**453**	117	29	599
Neither	99	**30**	10	139
Disagree	62	29	**18**	109
Total	614	176	57	847

**Surgeons**				
Agree	**282**	52	30	364
Neither	77	**30**	8	115
Disagree	96	30	**37**	163
Total	455	112	75	642

**Other hospital specialists**				
Agree	**924**	166	72	1162
Neither	103	**42**	19	164
Disagree	78	30	**29**	137
Total	1105	238	120	1463

*Notes: Wilcoxon (W) and marginal homogeneity (MH) test results were as follows. W: GPs P*<*0.001; hospital medical specialists P* = *0.004; surgeons P*<*0.001; other hospital P* = *0.29. MH: GPs P*<*0.001; hospital medical specialists P* = *0.007; surgeons P*<*0.001; other hospital P* = *0.19.*
